# TRPV1–STAT3 signaling as a driver of lethal pathology during pneumococcal nose-to-brain invasion

**DOI:** 10.1128/spectrum.01320-26

**Published:** 2026-09-15

**Authors:** Hideki Sakatani, Ryo Ueda, Masamitsu Kono, Taku Fujishiro, Masayoshi Hijiya, Yohei Morita, Kohei Kawabata, Takuma Okada, Kazuya Mizobata, Shizuya Saika, Muneki Hotomi

**Affiliations:** 1 Department of Otorhinolaryngology-Head and Neck Surgery, Wakayama Medical University 13145, Wakayama, Japan; 2 Department of Ophthalmology, Wakayama Medical University 13145, Wakayama, Japan; Ludwig-Maximilians-Universitat Munchen Pettenkofer Institute, München, Germany

**Keywords:** transient receptor potential vanilloid 1, signal transducer and activator of transcription 3, invasive pneumococcal diseases, *Streptococcus pneumoniae*, spatial transcriptome

## Abstract

Nose-to-brain infection represents a non-hematogenous route by which pathogens invade the central nervous system via neural pathways. The olfactory and trigeminal systems are implicated in this process, but the molecular mechanisms underlying lethal pathology remain incompletely understood. We hypothesized that trigeminal TRPV1 and downstream STAT3 signaling contribute to disease progression during pneumococcal nose-to-brain infection. A murine intranasal infection model using *Streptococcus pneumoniae* was established with methimazole-induced olfactory epithelial damage and/or resiniferatoxin-mediated trigeminal TRPV1 ablation. We assessed bacterial burden and survival. Spatial transcriptomic analysis of coronal head sections containing the nasal cavity and olfactory bulb was performed to identify region-specific transcriptional responses. Olfactory epithelial damage significantly increased pneumococcal invasion into the olfactory bulb, but ablation of trigeminal TRPV1 improved survival. Spatial transcriptomic analysis identified a distinct transcriptional cluster enriched in methimazole-treated mice that was localized to the olfactory epithelium and adjacent olfactory bulb. The cluster exhibited a STAT3-centered transcriptional regulatory network and enrichment of inflammatory and tissue remodeling pathways. These features suggest the formation of a localized inflammatory niche associated with olfactory epithelial damage. Olfactory epithelial damage and trigeminal TRPV1 activation were suggested to exacerbate nose-to-brain infection cooperatively through STAT3-mediated inflammatory signaling. This TRPV1-STAT3 axis could be a key mechanism linking sensory neuronal activation to lethal neuroinflammation. Targeting this pathway could provide a novel therapeutic strategy for preventing severe central nervous system complications associated with respiratory infections.

**IMPORTANCE:**

Pneumococcal infection can spread from the nasal cavity to the brain via neural pathways, leading to severe and often fatal disease. However, the mechanisms that drive disease severity have been poorly understood. Using a mouse model, we show that damage to the olfactory epithelium establishes a local inflammatory niche, while activation of the trigeminal nerve amplifies deleterious immune responses. We identify a key signaling pathway that links sensory nerve activation to excessive inflammation in the brain. These findings suggest that targeting host responses, rather than the bacteria alone, may mitigate severe complications of pneumococcal infection. This study provides new insight into the contribution of sensory systems to infection outcomes and highlights potential strategies for therapeutic intervention.

## INTRODUCTION

Central nervous system (CNS) tissues are normally protected by the blood-brain barrier. However, some pathogens can directly invade the CNS from the nasal cavity via non-hematogenous routes (1, 2). The cranial bones separating the brain from the nasal cavity contain numerous foramina through which nerve fibers pass. These anatomical structures provide potential pathways for non-hematogenous nose-to-brain infection (3, 4). CNS infections are life-threatening conditions with poor clinical outcomes and can result in functional impairments such as motor, sensory, or cognitive deficits (5, 6). Elucidating the molecular mechanisms underlying nose-to-brain infection is therefore thought to be an important step toward better understanding of infectious diseases of the CNS and identifying novel therapeutic targets.

The sensory nervous system innervating the nasal cavity plays a critical role in nose-to-brain infection, with the olfactory nerve and the trigeminal nerve as its principal components. Olfactory receptor neurons form an epithelial barrier in the nasal cavity and can physically restrict pathogen entry into the CNS via the nose-to-brain route (7). In contrast, the trigeminal neurons detect potentially harmful stimuli and initiate protective responses that facilitate the clearance of foreign substances from the nasal cavity (8, 9). These mechanisms comprise a form of sensory immunology in which sensory neurons modulate immune responses (10).

Transient receptor potential vanilloid (TRPV) channels are a family of ion channels that detect noxious and inflammatory stimuli (11, 12). They also participate in the regulation of immune responses and neuroinflammation (13). Within this subfamily, TRPV1 is expressed in both olfactory and trigeminal sensory neurons (14).

Despite the clinical use of pneumococcal polysaccharide vaccines, *Streptococcus pneumoniae* remains a leading cause of invasive infections, especially in vulnerable populations such as younger children and elderly people (15–17). We previously used a murine model to demonstrate that activation of trigeminal TRPV1 during nose-to-brain infection induces excessive expression of IL-6 and TNF-α, thereby contributing to the development of lethal pathology (14). However, the molecular mechanisms underlying how TRPV1 activation leads to these inflammatory responses remain incompletely understood. Our previous findings suggested the involvement of signal transducer and activator of transcription 3 (STAT3). As the JAK/STAT3 signaling pathway plays a central role in inflammatory responses associated with infection and tissue injury (18), we hypothesized that activation of trigeminal TRPV1 would exacerbate infection via the STAT3 signaling pathway.

In this study, we employ a mouse model of intranasal pneumococcal infection and conduct spatial transcriptomic analysis to investigate the molecular mechanisms driving lethal pathology during nose-to-brain infection.

## RESULTS

### Olfactory epithelial injury facilitates intracranial invasion, while trigeminal TRPV1 activation contributes to lethality

**Fig 1:**
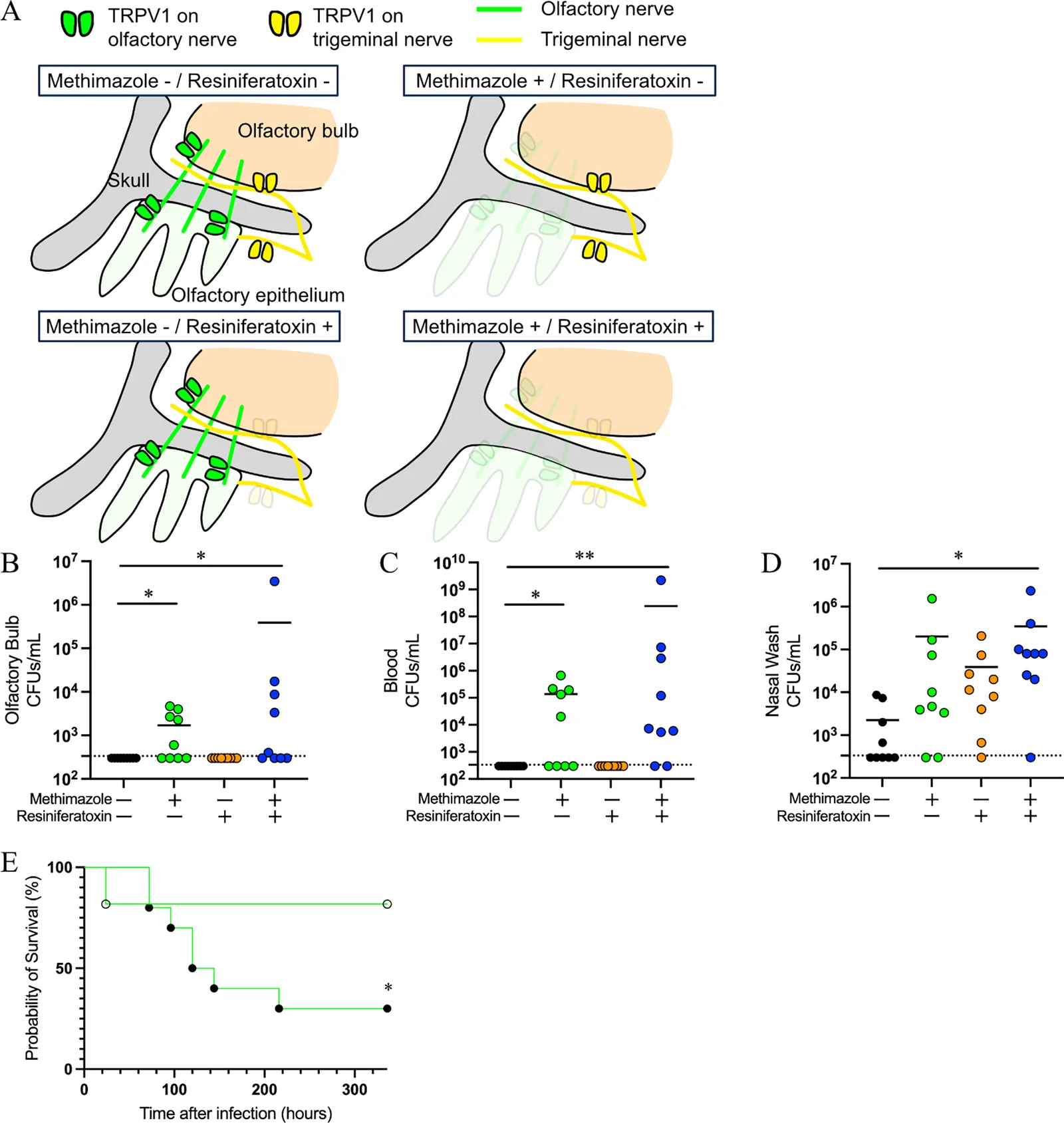
Olfactory epithelial injury facilitates intracranial invasion, while trigeminal TRPV1 contributes to lethality (**A**) Schematic representation of the experimental model. Olfactory sensory neurons extend axons from the olfactory epithelium to the olfactory bulb, while trigeminal nerve fibers innervate both the nasal cavity and intracranial tissues. TRPV1 is expressed in both neurons. Methimazole induces degeneration of the olfactory epithelium and disrupts TRPV1-expressing olfactory neurons. Resiniferatoxin induces functional impairment of TRPV1 in trigeminal nerve fibers. (**B–D**) WT mice were intranasally infected with *S. pneumoniae*. Bacterial burdens were assessed 72 h after infection in the olfactory bulb (**B**), blood (**C**), and nasal lavage fluid (**D**). Each dot represents an individual mouse (*n* = 9), and horizontal bars indicate the median. (**E**) Survival analysis of methimazole-pretreated WT and TRPV1 knockout mice following intranasal *S. pneumoniae* infection (*N* = 10 per group). Black circles indicate WT mice, and open circles indicate TRPV1 knockout mice. Statistical analyses were performed using the Kruskal-Wallis test followed by Dunn’s multiple-comparison test for (**B–D**) and the log-rank test for (**E**) *P < 0.05; **P < 0.01.

Olfactory receptor neurons form an epithelial barrier that separates the nasal cavity from the brain and can be selectively damaged by methimazole (19). In contrast, resiniferatoxin selectively ablates TRPV1-positive trigeminal neurons in the nasal cavity (20) (Fig. 1A). Six- to eight-week-old wild-type (WT) mice were randomly assigned to four experimental groups: a group without treatment (control group), a group treated with methimazole (MZ group), a group treated with resiniferatoxin (RTX group), and a group treated with methimazole plus resiniferatoxin (MZ-RTX group). Mice were intranasally infected with *S. pneumoniae* serotype 6A. We assessed the effects of olfactory epithelial damage and trigeminal TRPV1 on bacterial dissemination 72 h after infection. Bacterial loads in the blood and olfactory bulb were significantly higher in the MZ and MZ-RTX groups than in the control group, but no significant difference was observed between the RTX and control groups (Fig. 1B and C). In addition, bacterial loads in nasal lavage fluid were significantly increased in the MZ-RTX group compared with the control group (Fig. 1D). Further assessment of survival in the MZ group revealed that WT mice (black circles) had significantly lower survival than TRPV1 knockout (KO) mice (open circles) (Fig. 1E). These results indicate that olfactory epithelial damage facilitates intracranial bacterial invasion, while trigeminal TRPV1 contributes to the fatal progression of infection.

### Quality assessment of spatial transcriptomic data

To investigate gene expression patterns in the intranasal pneumococcal infection model, we performed spatial transcriptomic analysis. First, to assess the quality of the spatial transcriptomic data, we examined the morphology of coronal head sections containing the nasal cavity and olfactory bulb by hematoxylin-eosin (HE) staining. Tissue sections from the control group and the MZ group were mounted on one slide (Fig. S1A). Tissue sections from the RTX group and the MZ-RTX group were mounted on another slide (Fig. S2A). To assess the quality of the spatial transcriptomic data, we examined the distribution of detected genes (nFeature_Spatial) and unique molecular identifiers (UMIs; nCount_Spatial) across the samples. In all samples, both the number of detected genes and UMI counts per spot exhibited broad distributions, which indicated that sufficient transcriptomic information was obtained for downstream analyses (Fig. S1B and C and Fig. S2B and C). The spatial distribution of these metrics was then mapped onto the tissue sections before and after quality filtering. Prior to filtering, nCount_Spatial and nFeature_Spatial were distributed across the entire capture area (Fig. S1D through G and Fig. S2D through G). After removing low-quality spots based on these metrics, the remaining spots were predominantly localized to tissue regions and aligned well with the underlying histological structures (Fig. S1H through K and Fig. S2H through K). We further examined the distribution of spots from each sample in the uniform manifold approximation and projection (UMAP) embedding. Spots from all experimental groups were broadly distributed across the UMAP space, and no apparent clustering bias associated with specific samples was observed (Fig. S3A through D). In addition, UMI counts and detected gene numbers were evenly distributed across the UMAP space without enrichment in specific clusters, indicating that the clustering patterns reflected biological differences rather than technical artifacts (Fig. S3E and F).

### Identification of transcriptional clusters by spatial transcriptomic analysis

**Fig 2:**
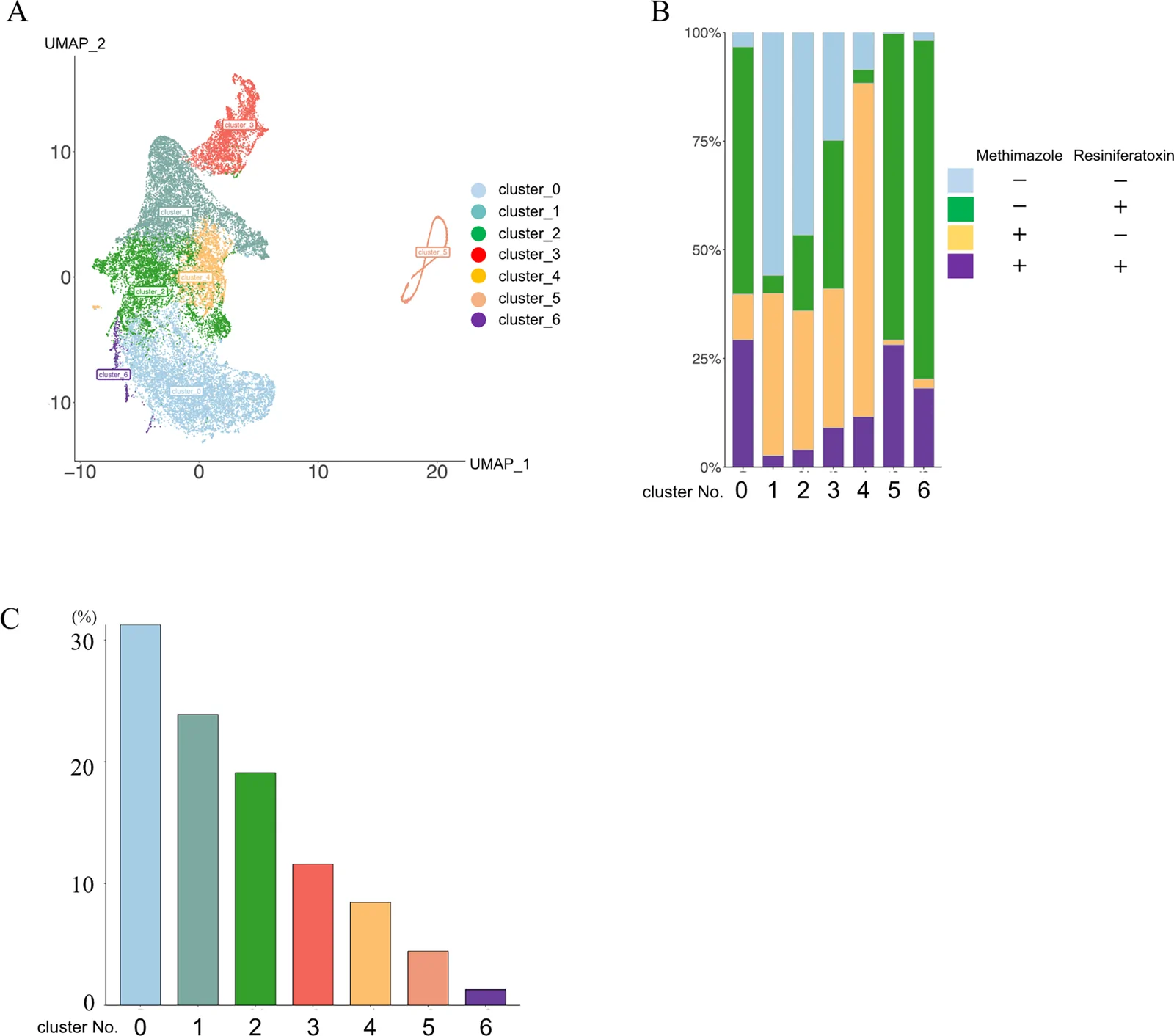
Identification of transcriptional clusters by spatial transcriptomic analysis (**A**) UMAP visualization of spatial transcriptomic spots derived from tissue sections. Spots were classified into seven transcriptional clusters (clusters 0–6) based on gene expression profiles. (**B**) Distribution of experimental groups across clusters. Bars represent the proportion of spots derived from each condition (control, MZ, RTX, and MZ-RTX group) within each cluster. (**C**) Relative abundance of each transcriptional cluster across all spatial transcriptomic spots. The percentage of spots assigned to each cluster is shown.

We performed clustering analysis based on spatial transcriptomic gene expression profiles. This analysis revealed seven transcriptional clusters (clusters 0–6) (Fig. 2A). Dimensionality reduction using UMAP showed that these clusters were clearly separated from one another, indicating the presence of multiple transcriptionally distinct cellular populations within the tissue. Cluster 4 contained a relatively higher proportion of spots from the MZ group (Fig. 2B). In contrast, clusters 1, 2, and 3 were broadly distributed across multiple experimental groups, suggesting that these clusters represent transcriptional populations shared under different conditions. Clusters 0, 5, and 6 contained relatively higher proportions of spots derived from the RTX group. Although cluster 4 comprised approximately 8% of all spatial transcriptomic spots, it was preferentially enriched in the MZ group (Fig. 2C).

### Spatial distribution of transcriptional clusters within nasal tissues

**Fig 3:**
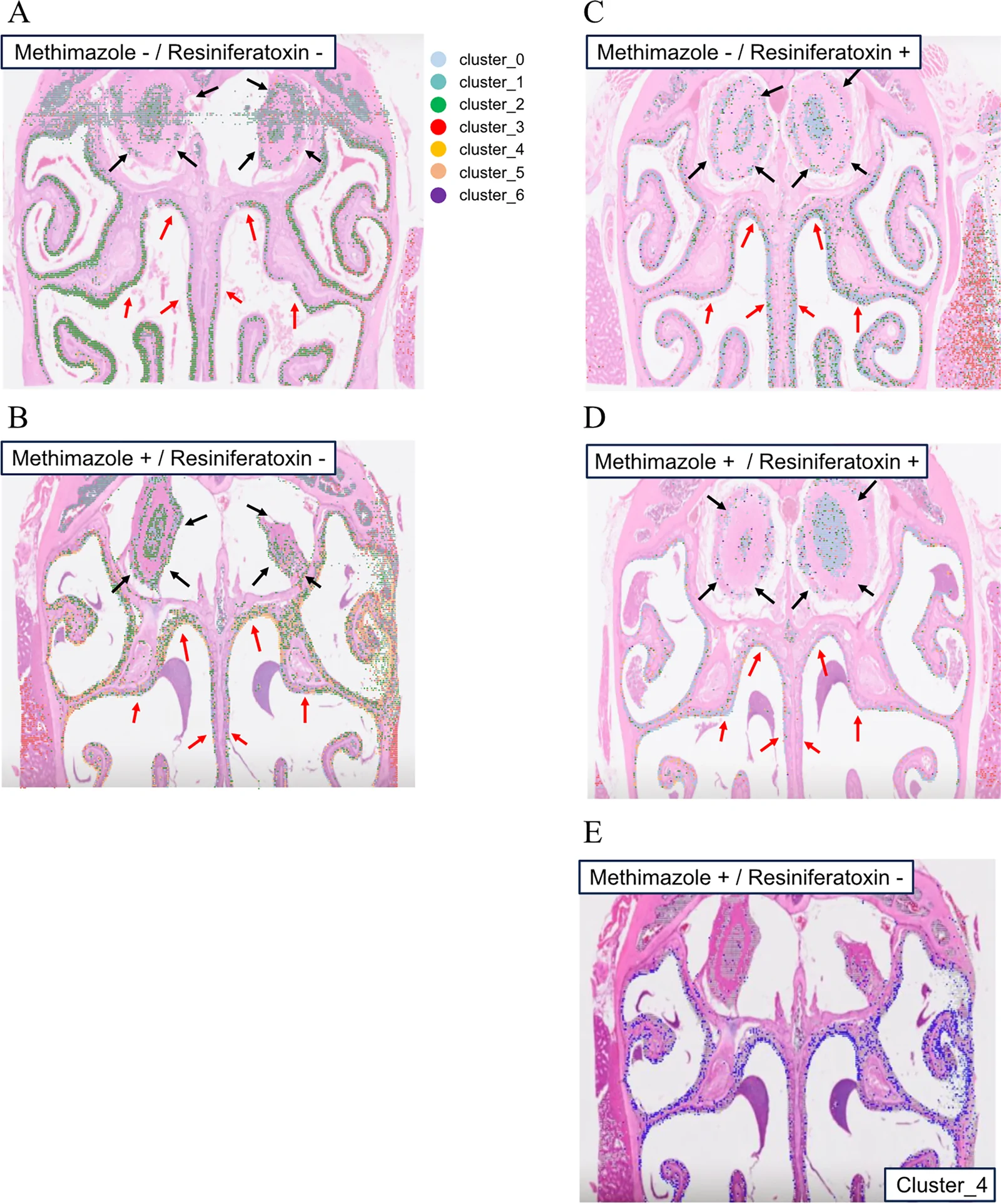
Spatial distribution of transcriptional clusters within nasal tissues (**A–D**) Spatial mapping of transcriptional clusters onto HE-stained coronal head sections from each experimental group. Colored dots represent spatial transcriptomic spots assigned to each cluster (clusters 0–6). Panels correspond to control (**A**), MZ (**B**), RTX (**C**), and MZ-RTX (**D**) groups. Black arrows indicate the olfactory bulb, and red arrows indicate the olfactory epithelium. (**E**) Spatial distribution of cluster 4 in the MZ group. Spots assigned to cluster 4 are highlighted, demonstrating enrichment in the olfactory epithelium and olfactory bulb.

We next mapped the spatial distribution of these transcriptional clusters onto the tissue sections (Fig. 3). Spatial transcriptomic spots were predominantly localized to the olfactory epithelium (red arrows) and the olfactory bulb (black arrows). The predominant clusters varied across experimental groups. In the control group, the major cluster was cluster 2 (Fig. 3A). In the MZ group, the predominant cluster was cluster 4 (Fig. 3B), whereas cluster 0 was dominant in both the RTX and MZ-RTX groups (Fig. 3C and D). To further characterize cluster 4, we examined its spatial distribution specifically in the MZ group. Cluster 4 was mainly localized to the olfactory epithelium and parts of the olfactory bulb based on spatial correspondence with HE-stained sections (Fig. 3E).

### Cluster-specific gene expression and functional characterization of cluster 4

**Fig 4:**
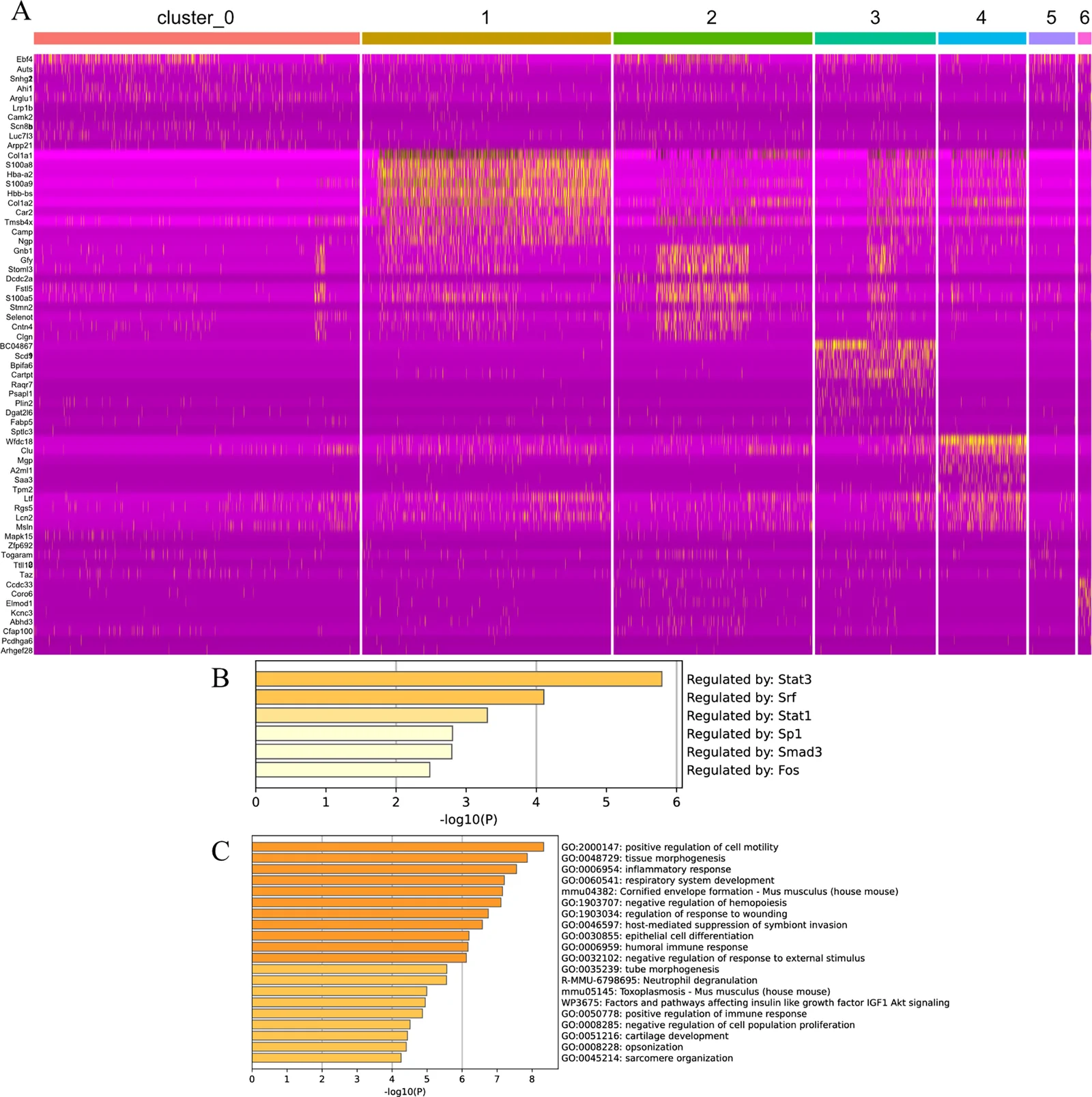
Cluster-specific gene expression and functional characterization of cluster 4 (**A**) Heatmap of top marker genes across transcriptional clusters (clusters 0–6). Rows represent genes, and columns represent spatial transcriptomic spots grouped by cluster identity. (**B**) Transcription factor enrichment analysis of cluster 4-associated genes. STAT3 showed the highest enrichment, followed by SRF, STAT1, SP1, SMAD3, and FOS. Bars represent –log10(P) values. (**C**) Gene Ontology biological process enrichment analysis of cluster 4-associated genes. Enriched terms include inflammatory response, positive regulation of cell motility, and tissue morphogenesis. Bars indicate statistical significance expressed as –log10(P).

Top marker genes for each cluster are shown in a heatmap in Fig. 4A. Clusters 1, 2, 3, and 4 exhibited relatively distinct transcriptional signatures compared with the other clusters. The top 100 marker genes for cluster 4 are listed in Table 1. The corresponding marker genes for the remaining clusters are provided in Table S1. The numbers of significantly enriched genes with an adjusted *P* value < 0.05 in clusters 0–6 were 19, >100, >100, 84, >100, 1, and 11, respectively. Clusters 1, 2, 3, and 4 were therefore selected for further downstream analyses. Cluster 4 showed the highest specificity for the MZ group in Fig. 2B. Transcription factor enrichment analysis identified STAT3 as the most strongly enriched transcription factor (Fig. 4B). Other transcription factors, including SRF, STAT1, SP1, SMAD3, and FOS, were also predicted to be transcriptional regulators of cluster 4 genes. Gene ontology enrichment analysis further showed that cluster 4 genes were significantly associated with biological processes such as positive regulation of inflammatory response, positive regulation of cell motility, and tissue morphogenesis (Fig. 4C). These results suggest that cluster 4 is characterized by transcriptional features associated with inflammatory responses and tissue remodeling. Notably, the enrichment of STAT3-associated transcriptional regulation suggests a potential link between trigeminal TRPV1 signaling and inflammatory responses during nose-to-brain infection. In the MZ group, clusters 1, 2, and 3 were mainly distributed within the cranial bone marrow, olfactory epithelium, and olfactory bulb, and masticatory muscle, respectively (black arrows, Fig. S4A, C and E). Gene ontology enrichment analysis suggested that cluster 1 reflected vascular or hematopoietic/immune-associated components within the cranial bone, whereas cluster 2 represented an olfactory epithelium-associated compartment shared across experimental conditions (Fig. S4B and D). Cluster 3 did not identify biologically meaningful pathways or tissue signatures (Fig. S4F). Compared with these clusters, cluster 4 may reflect a transcriptional program that is more specifically associated with the MZ group.

**TABLE 1:** Top 100 marker genes identified in cluster 4

Cluster number		Gene	Log2 fold change	Expressing cells (%)	Expressing cells (others) (%)	Adjusted P value
4	1	Wfdc18	5.288287441	0.585	0.027	0
	2	Clu	2.697918005	0.32	0.062	0
	3	Mgp	4.59807309	0.173	0.011	0
	4	A2ml1	6.18600448	0.105	0.002	0
	5	Saa3	5.627819552	0.101	0.002	0
	6	Tpm2	4.364524444	0.093	0.006	4.16525630822696e-273
	7	Ltf	2.121023495	0.261	0.065	2.49938745731014e-256
	8	Rgs5	1.925894584	0.179	0.047	1.95900768844678e-154
	9	Lcn2	2.120114491	0.175	0.048	3.80073062719663e-146
	10	Msln	1.716871494	0.147	0.036	5.52238607625542e-139
	11	Igfbp5	1.454309318	0.238	0.088	1.25208961495401e-124
	12	Apod	1.278560606	0.26	0.114	4.03358743512371e-97
	13	Tmem176b	1.940231782	0.101	0.024	1.78582418030072e-94
	14	Pigr	2.321094171	0.055	0.009	2.75275301963975e-77
	15	Gpx3	2.694094026	0.071	0.016	7.90491807076326e-74
	16	Igfbp7	1.676533814	0.118	0.038	3.62057045030452e-73
	17	Cbr2	1.236613394	0.179	0.084	4.42202630499494e-55
	18	Prdx6	2.338242952	0.04	0.008	2.04055290081781e-47
	19	C1s1	1.887936656	0.052	0.013	1.33770807042935e-45
	20	Chil1	2.510397317	0.025	0.003	2.93438455485615e-45
	21	Ly6e	1.678464082	0.066	0.019	1.17623587460699e-43
	22	Bpifa1	2.858294021	0.022	0.003	1.06173681272555e-42
	23	Ifitm1	2.19309049	0.039	0.008	3.14055314513676e-41
	24	Reg3g	2.237737352	0.034	0.007	9.84516794557929e-40
	25	Acta2	1.759217308	0.053	0.015	6.6645906624896e-38
	26	H2-K1	1.277359827	0.084	0.031	2.07353617747187e-37
	27	Serpina3n	2.558141449	0.03	0.006	4.71788197961367e-36
	28	Itgb1	1.454078619	0.078	0.029	7.40302100238869e-34
	29	Cd74	1.504445911	0.078	0.03	8.10608564803376e-34
	30	Wfdc2	1.587111377	0.042	0.011	1.60717884440209e-33
	31	Rbp1	1.932078088	0.038	0.01	5.6196573404616e-32
	32	S100a11	1.435614432	0.07	0.026	3.97296647372682e-31
	33	Gbp2	2.551016356	0.017	0.002	8.86315058033112e-30
	34	Lbp	1.988270988	0.029	0.006	9.41356100249522e-30
	35	Tagln	1.533784804	0.054	0.018	2.06648319192923e-28
	36	Igfbp4	1.283389	0.079	0.033	5.43255886598149e-27
	37	Ifitm2	1.466037891	0.068	0.027	1.20390139861215e-26
	38	Cxcl9	3.343470038	0.011	0.001	9.24022756061442e-25
	39	Ly6a	2.15112072	0.019	0.004	5.77503866174667e-23
	40	Lypd2	1.573814596	0.041	0.013	9.09269214120332e-23
	41	Inmt	1.218439802	0.056	0.021	1.02440552480938e-22
	42	Gusb	1.157093308	0.059	0.023	7.61002329906965e-22
	43	Aldh1a2	1.49159273	0.042	0.014	2.51471451863952e-21
	44	Ifitm3	1.760533853	0.027	0.007	3.77074002541634e-21
	45	Sult1c1	1.053919258	0.099	0.051	9.05582527922425e-21
	46	Anxa1	1.451570841	0.038	0.012	4.60467521818068e-19
	47	Muc1	1.986133757	0.015	0.003	6.71814618830042e-19
	48	Hspb8	1.90768158	0.021	0.005	1.13882164697211e-18
	49	Tceal9	1.292181234	0.054	0.022	1.42608447117444e-18
	50	Fetub	1.302878803	0.06	0.026	8.07658080614545e-18
	51	Vim	1.142633576	0.089	0.046	1.15035223876188e-17
	52	Krt18	1.483397228	0.04	0.015	2.73370113516972e-17
	53	Serping1	1.658325963	0.028	0.008	7.14728809765829e-17
	54	Krt8	1.248913133	0.038	0.013	1.79741840088111e-16
	55	Sprr1a	1.733995923	0.014	0.003	2.28395502053527e-16
	56	Krt19	1.620676188	0.022	0.006	8.55667285034325e-16
	57	Cxcl17	2.166972922	0.012	0.002	7.27139519859003e-14
	58	Ctsh	2.164645282	0.018	0.005	1.85996377314408e-13
	59	Cstb	1.65864812	0.019	0.005	1.75072587182574e-12
	60	Mdk	1.413926136	0.02	0.006	1.86594311323483e-12
	61	Gsta4	1.32209465	0.024	0.008	2.07625090788488e-12
	62	Stat3	1.307978005	0.025	0.008	2.13410771426119e-12
	63	Serpinh1	1.298237514	0.039	0.016	2.33166396825884e-12
	64	H2-Eb1	1.431153977	0.033	0.013	4.26648362456199e-12
	65	Kcnj15	1.654523499	0.016	0.004	1.16337495515605e-11
	66	Sftpd	1.562727238	0.019	0.005	1.69575636619066e-11
	67	Six1	1.261058241	0.031	0.011	1.72996657177874e-11
	68	St14	1.874614949	0.016	0.004	2.3292007055429e-11
	69	Psmd13	1.526875767	0.031	0.012	3.83104743012717e-11
	70	Igf2	1.475149933	0.027	0.009	3.94788337941948e-11
	71	Pglyrp1	2.116950969	0.017	0.005	4.17908826821312e-11
	72	Cfh	1.46796984	0.026	0.009	2.37997755866571e-10
	73	Itgb4	1.745468101	0.014	0.003	2.70202284684941e-10
	74	Oasl2	1.724161766	0.013	0.003	4.21145932276681e-10
	75	Cnn3	1.398910234	0.037	0.016	5.07911471464528e-10
	76	Postn	1.563869599	0.024	0.008	6.96470112809585e-10
	77	Csrp2	1.376932393	0.024	0.008	1.132597577516e-09
	78	Six2	1.459726584	0.022	0.007	1.14177436435948e-09
	79	Bgn	1.108864338	0.051	0.025	1.23781026660895e-09
	80	Tmem176a	1.33910365	0.023	0.008	1.36830858268627e-09
	81	Krt5	1.245114	0.024	0.009	1.62489443027528e-09
	82	Ccn2	1.321320503	0.025	0.009	1.63222457045451e-09
	83	Nt5dc2	1.899875781	0.014	0.004	2.18907294918973e-09
	84	Plat	1.535279862	0.019	0.006	2.70725818253592e-09
	85	Irgm1	1.858735674	0.021	0.007	3.46098785956392e-09
	86	Col4a1	1.274404976	0.028	0.011	4.02409904274029e-09
	87	Pebp1	1.285899609	0.028	0.011	1.60731861427031e-08
	88	Lamc1	1.3512715	0.024	0.009	1.7797286341073e-08
	89	Cebpd	1.542374681	0.018	0.006	1.91103367407234e-08
	90	Clic6	1.112918083	0.027	0.011	2.25987387250562e-08
	91	Mxra8	1.471552053	0.023	0.008	2.764028431425e-08
	92	Eppk1	1.474806119	0.014	0.004	3.3572861118322e-08
	93	Smdt1	1.299114815	0.034	0.015	4.26414113696563e-08
	94	Pdpn	2.303628465	0.01	0.002	6.55619127331601e-08
	95	Ier3	1.693366612	0.017	0.006	1.05205583822675e-07
	96	H2-Ab1	1.313395245	0.023	0.009	1.16301260085236e-07
	97	Anxa2	1.688807513	0.023	0.009	1.2135544994227e-07
	98	Cldn7	1.74145592	0.013	0.003	1.34639200252038e-07
	99	Celsr1	1.28409487	0.017	0.005	1.57928447957661e-07
	100	Nr2e1	1.789802432	0.013	0.004	2.99354431315286e-07

### Validation of STAT3-associated signaling in methimazole-treated mice

**Fig 5:**
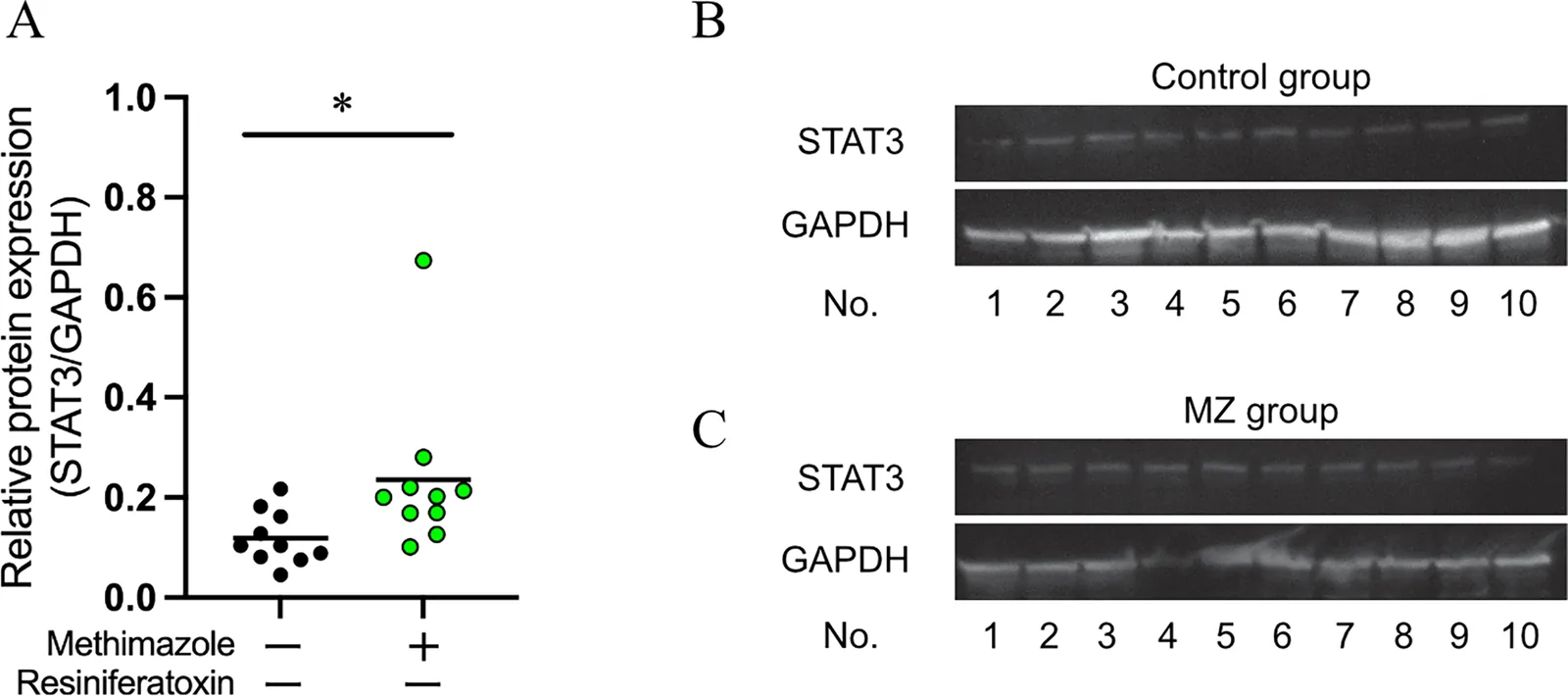
Validation of STAT3-associated signaling by Western blot analysis Western blot analysis of total STAT3 expression in olfactory bulb homogenates collected 72 h after intranasal *S. pneumoniae* infection. (**A**) Quantification of STAT3 expression normalized to GAPDH. Full STAT3 immunoblots with corresponding GAPDH controls from the control group and MZ group are shown in panels **B and C**, respectively. Each dot represents an individual mouse (*n* = 10 per group), and horizontal bars indicate the median for panel **A**. Each lane represents an individual mouse for panels **B and C**. Statistical analysis was performed using the Mann–Whitney U test. *P < 0.05.

**Fig 6:**
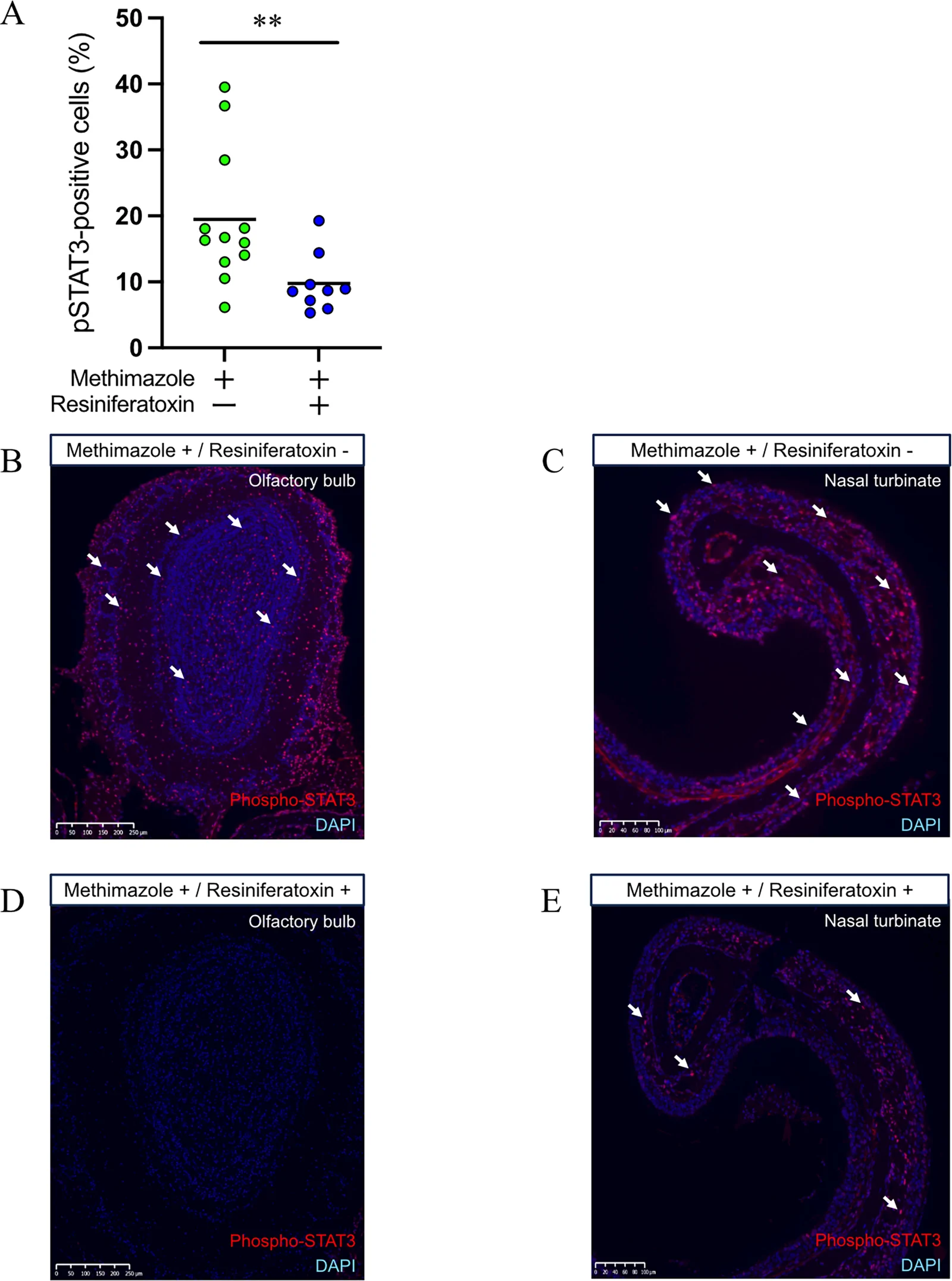
Validation of STAT3-associated signaling by phospho-STAT3 immunofluorescence staining Phospho-STAT3 immunofluorescence staining of coronal head sections collected 72 h after intranasal S. pneumoniae infection. (**A**) Quantification of phospho-STAT3-positive cells expressed as the percentage of DAPI-positive cells in the combined bilateral olfactory bulbs and nasal turbinates. Representative immunofluorescence images of the olfactory bulb (**B and D**) and nasal turbinate (**C and E**) from the MZ (**B and C**) and MZ-RTX (**D and E**) groups. Phospho-STAT3-positive cells are shown in red, nuclei are counterstained with DAPI (blue), and white arrows indicate phospho-STAT3-positive cells. Each dot represents an individual mouse (MZ, *n* = 12; MZ-RTX, *n* = 9), and horizontal bars indicate the median for (**A**). Statistical analysis was performed using the Mann–Whitney U test. **P < 0.01. Scale bars, 250 μm (**B and D**) and 100 μm (**C and E**).

To further validate the STAT3-associated transcriptional signature identified by spatial transcriptomic analysis, we performed Western blot analysis of olfactory bulb homogenates collected 72 h after *S. pneumoniae* infection. Total STAT3 expression normalized to GAPDH was significantly increased in the MZ group (Fig. 5A through C). These findings support the enrichment of STAT3-associated transcriptional programs identified by spatial transcriptomic analysis. Phospho-STAT3 immunostaining was subsequently performed to assess the association between TRPV1 signaling and STAT3-associated responses. The number of phospho-STAT3-positive cells was significantly lower in the MZ-RTX group than in the MZ group (Fig. 6A through E). These findings suggest that trigeminal TRPV1 dysfunction is associated with attenuation of STAT3-related signaling following olfactory epithelial injury and pneumococcal infection.

## DISCUSSION

We investigated the roles of olfactory epithelial damage and trigeminal TRPV1 in the pathogenesis of pneumococcal nose-to-brain infection. Disruption of olfactory epithelium by methimazole treatment appeared to be a key factor in facilitating nose-to-brain invasion. In contrast, trigeminal TRPV1 apparently contributed less to bacterial entry itself and more to the host responses following infection. Notably, survival data suggest that disease severity cannot be explained solely by bacterial burden, but that it involves trigeminal TRPV1-dependent inflammatory responses that drive lethal pathology. These findings are consistent with the results of our previous work and other studies that have described neuro-immune interactions during infection (14, 21).

Olfactory receptor neurons have been shown to exhibit a remarkable capacity for regeneration throughout life (22). The increase in cluster 4 observed in the MZ group in the current study may reflect a transcriptional state associated with olfactory neuron regeneration. Regenerative processes in neural tissues are also accompanied by dynamic changes in the local immune cell populations and cytokine milieu (23). Methimazole induces broad injury to the olfactory epithelium, including sustentacular cells, Bowman’s gland cells, and olfactory receptor neurons, and it is known to trigger subsequent regenerative responses (24). Although cluster 4 may reflect a transcriptional state associated with olfactory epithelial regeneration, its reduced abundance following RTX treatment suggests that this transcriptional program may not solely reflect methimazole-induced injury and regeneration alone. Rather, it may also be associated with TRPV1-dependent modulation of the local immune environment during nose-to-brain infection.

The olfactory epithelium is a neural tissue that is directly exposed to the external environment and undergoes immune responses and epithelial remodeling upon pathogen exposure (25). Olfactory nerve fibers extend to the olfactory bulb through the cribriform plate, and both these fibers and the foramina they traverse can serve as potential entry routes for pathogens into the CNS (4). In this analysis, the same transcriptional clusters were distributed across the olfactory epithelium and olfactory bulb, and the predominant clusters differed among experimental groups. Notably, cluster 0, which predominated in the RTX and MZ-RTX groups, contained only 19 marker genes with an adjusted *P* value < 0.05, and this is markedly fewer than the >100 genes identified in cluster 4. These findings suggest that the transcriptional program represented by cluster 4 is associated with TRPV1-dependent regulation via trigeminal signaling.

In this study, genes characteristic of cluster 4 were strongly associated with a STAT3-centered transcriptional regulatory network. STAT3 is a key transcription factor in the JAK/STAT sig

naling pathway. It is activated by inflammatory cytokines such as IL-6 and TNF-α, and is involved in inflammatory responses, tissue repair and immune regulation (26). Gene ontology analysis further indicated activation of JAK/STAT signaling in the olfactory epithelium and olfactory bulb. STAT3 can also drive excessive host responses (27, 28). Whether STAT3 activation is beneficial or detrimental depends on the magnitude of activation, the responding cell types, and the local cytokine milieu (29, 30). In this context, increased bacterial invasion following olfactory epithelial damage may induce excessive STAT3 activation, thereby contributing to lethal outcomes in mice. These localized transcriptional responses often develop within spatially restricted inflammatory microenvironments (31). Our findings suggest that cluster 4 may represent a spatially localized transcriptional program associated with inflammatory responses within the olfactory epithelium and adjacent olfactory bulb following infection. The spatial transcriptomic bins contain multiple cell types, so cluster 4 likely reflects mixed cellular populations rather than a single defined lineage, but we could not determine the precise cellular composition from the current data set.

Western blot analysis demonstrated increased STAT3 expression in the MZ group and supported the spatial transcriptomic identification of a STAT3-centered transcriptional program. The reduction of phospho-STAT3-positive cells in the MZ-RTX group further supports an association between trigeminal TRPV1 signaling and STAT3-associated responses. However, these findings should be interpreted as evidence of an association between trigeminal TRPV1 signaling and STAT3-associated responses rather than as definitive proof of a causal TRPV1–STAT3 signaling pathway.

In the CNS, TRPV1 has been reported to activate the JAK/STAT3 signaling pathway and induce neuroinflammation (32). In pneumococcal nose-to-brain infection, activation of trigeminal TRPV1 induces IL-6 and TNF-α expression and contributes to lethal inflammatory responses (14). The STAT3-centered transcriptional network may therefore represent a downstream component of TRPV1-dependent inflammatory signaling.

Taken together, our findings suggest that olfactory epithelial damage creates a spatially localized transcriptional program associated with inflammatory responses, in which trigeminal TRPV1 signaling may be linked to a STAT3-centered transcriptional network during nose-to-brain infection. These transcriptional responses may contribute to disease severity and lethal pathology. Olfactory dysfunction is a common and often refractory condition with diverse etiologies (33–35), and its prevalence is expected to increase in aging populations. Impaired olfaction increases susceptibility to nose-to-brain infection (14), so targeting TRPV1 or STAT3 signaling may, from a senso-immunology perspective, represent a potential strategy to mitigate excessive inflammatory responses and disease severity.

Several limitations should be acknowledged in relation to this study. Methimazole treatment may induce broader olfactory epithelial damage and subsequent regenerative responses, whereas resiniferatoxin treatment may affect TRPV1-positive sensory pathways beyond trigeminal neurons. The observed phenotypes may therefore partially reflect epithelial remodeling and broader sensory modulation in addition to TRPV1-dependent mechanisms.

## MATERIAL AND METHODS

### Mice

Male mice aged 6–8 weeks were used in all experiments. Wild-type (WT) C57BL/6J mice were obtained from Charles River Laboratories Japan, and TRPV1-deficient mice (TRPV1 knockout, TRPV1 KO; B6.129 X 1-Trpv1^tm1Jul/J^; JAX 003770) on the same genetic background were purchased from the Jackson Laboratory. The knockout mice lack functional TRPV1 due to targeted disruption of the *trpv1* gene (36). All animals were maintained under standard housing conditions at the animal facility at Wakayama Medical University.

### Methimazole- and resiniferatoxin-driven TRPV1 dysfunction model

To ablate TRPV1-expressing olfactory sensory neurons, methimazole (75 mg/kg; FUJIFILM Wako Pure Chemicals, Osaka, Japan) was administered intraperitoneally 2 days prior to *S. pneumoniae* infection. Methimazole is known to selectively damage the olfactory epithelium, including olfactory receptor neurons, sustentacular cells, and Bowman’s gland cells, with peak damage occurring between 24 h and 7 days after administration in rodents (37, 38). To induce long-term functional impairment of TRPV1-positive sensory pathways, including trigeminal neurons, resiniferatoxin was administered intraperitoneally at doses of 80, 100, and 150 ng/g body weight for three consecutive days as previously described, 30 days before *S. pneumoniae* infection (14, 39).

### Preparation of *S. pneumoniae* and intranasal infection

*S. pneumoniae* serotype 6A strain, P2431, was kindly provided by J. N. Weiser (New York University). This encapsulated strain is virulent and has been reported to induce sepsis following intranasal infection (40). Bacteria were grown in tryptic soy broth (Becton Dickinson, Franklin Lakes, NJ) at 37°C to mid-exponential phase (OD600 ≈ 0.5). Cultures were subsequently washed and resuspended in sterile phosphate-buffered saline (PBS) to the desired concentration. Bacterial density was confirmed by plating serial 10-fold dilutions in triplicate on sheep blood agar plates, followed by overnight incubation at 37°C in 5% CO₂. For infection, mice were intranasally inoculated with 10 μL of bacterial suspension containing 1.0 × 10³ to 1.3 × 10³ colony-forming units (CFUs)/μL.

### Quantification of *S. pneumoniae* in tissues

At 72 h after intranasal infection, mice were euthanized with isoflurane, and we collected nasal lavage fluid, blood, and olfactory bulbs. Nasal lavage samples were obtained by intratracheal instillation of 200 μL sterile PBS. Olfactory bulbs were excised, rinsed in sterile PBS, and homogenized in 1 mL PBS (14). Aliquots (20 μL) of each sample were serially diluted and plated on sheep blood agar plates. After overnight incubation at 37°C in 5% CO₂, CFUs of *S. pneumoniae* were enumerated. The limit of detection was 6.66 × 10² CFUs/mL.

### Assessment of survival after intranasal infection

WT and TRPV1 knockout mice were pretreated with methimazole, followed by intranasal *S. pneumoniae* infection as described above. After infection, mice were monitored for survival at 24-h intervals up to 336 h. Mortality was recorded at each time point.

### Preparation of tissue sections

At 72 h after *S. pneumoniae* infection, mice were euthanized and whole heads were harvested and fixed in 4% paraformaldehyde. Samples were subsequently decalcified, dehydrated, and embedded in paraffin. Coronal sections of the nasal cavity were cut from paraffin blocks. Sections at the level of the olfactory bulb, corresponding to the mid-orbital plane, were selected for spatial transcriptomic analysis.

### Spatial transcriptomic analysis

Spatial transcriptomic analysis was performed using coronal head sections from one representative mouse per group (control, MZ, RTX, and MZ-RTX). Library preparation and sequencing were conducted by CyberomiX (Kyoto, Japan) using the Visium HD platform (10x Genomics) (41). Two tissue sections were mounted on each slide: sections from the control and MZ groups were placed on slide D1, whereas those from the RTX and MZ-RTX groups were placed on slide A1. Following tissue permeabilization, reverse transcription, and library preparation, spatially barcoded cDNA libraries were generated and sequenced to obtain gene expression data. The data were binned at a resolution of 16 μm, and gene expression counts were calculated for each bin. Quality control was performed based on the number of detected genes (nFeature_Spatial) and total RNA counts (nCount_Spatial). Baseline distributions of nFeature_Spatial and nCount_Spatial differed between slides, so slide-specific quality control thresholds were applied to remove low-quality bins while preserving comparable transcriptomic distributions across samples. Bins with nFeature_Spatial and nCount_Spatial values <50 for slide D1 or <25 for slide A1 were excluded from further analysis. Quality control metrics and cluster distributions for each slide are shown in Fig. S1 through S3. Filtered data were analyzed in R using the Seurat package. Gene expression matrices from all samples were integrated into a single Seurat object, followed by log normalization and scaling. Dimensionality reduction was performed using principal component analysis, and data were visualized using UMAP (42). Clustering was conducted using a graph-based algorithm implemented in Seurat with a resolution parameter of 0.7. Cluster-specific marker genes were identified using the FindAllMarkers function, and top markers were visualized as heatmaps. Spatial distributions of clusters were mapped onto tissue sections using the SpatialPlot function, and we compared differences in spatial organization and cluster composition among experimental groups.

### Functional enrichment analysis

Functional enrichment analysis was performed using Metascape (https://metascape.org) (43). The top 100 differentially expressed genes in cluster 1–4 were used as input for Gene Ontology biological process enrichment analysis and transcription factor enrichment analysis. Enriched terms were ranked based on statistical significance (−log10 *P* value). Statistical significance was calculated using a hypergeometric test with multiple testing correction implemented in Metascape.

### Phospho-STAT3 immunofluorescence staining

Paraffin-embedded coronal head sections (prepared as described above) were used for immunofluorescence staining. Sections were incubated with a rabbit monoclonal antibody against phospho-STAT3 (Tyr705) (1:75 dilution; Cell Signaling Technology, Danvers, MA, USA) and counterstained with 4′,6-diamidino-2-phenylindole (DAPI). Immunofluorescence staining was performed by Morpho Technology (Sapporo, Japan). Images were acquired using an NDP.view2 digital slide scanner (Hamamatsu Photonics, Hamamatsu, Japan). Quantitative image analysis was performed using QuPath software (University of Edinburgh, Edinburgh, UK). Based on the spatial distribution of cluster 4 identified by spatial transcriptomic analysis, phospho-STAT3-positive and DAPI-positive cells were quantified in the bilateral olfactory bulbs and nasal turbinates. The percentage of phospho-STAT3-positive cells was calculated by dividing the total number of phospho-STAT3-positive cells by the total number of DAPI-positive cells across these regions.

### Western blot analysis

At 72 h after *S. pneumoniae* infection, olfactory bulb homogenates were collected, and total protein was extracted using the Minute Total Protein Extraction Kit (Invent Biotechnologies, Plymouth, MN) supplemented with Halt Protease and Phosphatase Inhibitor Cocktail (Thermo Fisher Scientific, Waltham, MA). Protein concentrations were determined, and samples were denatured at 95°C for 5 min. Equal amounts of protein (30 μg per lane) were separated on 4%–20% Mini-PROTEAN TGX Stain-Free gels (Bio-Rad, Hercules, CA) using Tris-glycine-SDS running buffer. Proteins were transferred onto nitrocellulose membranes (Amersham Protran NC, 0.2 μm; Cytiva, Marlborough, MA) using a Trans-Blot SD semi-dry transfer system (Bio-Rad). Membranes were blocked with Tris Buffered Saline with Tween20 (TBS-T; Takara, Shiga, Japan) containing 1% bovine serum albumin (BSA; Wako, Osaka, Japan) and incubated with a primary antibody against STAT3 (1:2,000) (Cell Signaling Technology, Danvers, MA) for 1 h at room temperature. After washing with TBS-T, membranes were incubated with HRP-conjugated anti-rabbit IgG secondary antibody (1:1,000; Cell Signaling Technology) for 1 h at room temperature. Protein bands were detected using enhanced chemiluminescence (ECL Prime; Cytiva) and visualized with a LuminoGraph II imaging system (ATTO, Tokyo, Japan). After chemiluminescent detection of STAT3, membranes were incubated with WB Stripping Solution (Nacalai Tesque, Kyoto, Japan) for 15 min, washed three times with TBS-T for 10 min each, and re-probed with an anti-GAPDH antibody (1:1,000; Cell Signaling Technology). Band intensities were quantified using ImageJ software (National Institutes of Health, Bethesda, MD). STAT3 signals were normalized to the corresponding GAPDH signals.

### Statistical analysis

Statistical analyses for infection experiments were performed using GraphPad Prism (version 10.0; GraphPad Software). Survival curves were compared using the log-rank test. Comparisons among multiple groups were conducted using the Kruskal-Wallis test followed by Dunn’s multiple-comparison test. A *P* value <0.05 was considered statistically significant.

Spatial transcriptomic data were analyzed using the Seurat package (version 5.2.0) in R (version 4.3.3). Dimensionality reduction was performed using UMAP. Unsupervised clustering was conducted using the Louvain algorithm implemented in Seurat. Differentially expressed genes were identified using the Wilcoxon rank-sum test, with *P* values adjusted for multiple comparisons using the Bonferroni method. Adjusted *P* values <0.05 were considered statistically significant.

Functional enrichment analyses, including Gene Ontology and transcription factor enrichment analyses, were performed using Metascape (https://metascape.org). Enrichment significance was calculated using a hypergeometric test with multiple testing correction implemented in the platform.

## Data Availability

Raw and processed spatial transcriptomic data generated in this study have been deposited in the Gene Expression Omnibus (GEO) under accession number GSE331225. All other datasets used and/or analyzed during the current study are included in the figures and supplemental figures.

## SUPPLEMENTAL MATERIAL

Fig. S1 spectrum.01320-26-s0001.tiff Quality control of spatial transcriptomic data (control and MZ groups).

Fig. S2 spectrum.01320-26-s0002.tiff Quality control of spatial transcriptomic data (RTX and MZ-RTX groups).

Fig. S3 spectrum.01320-26-s0003.pdf Distribution of spatial transcriptomic spots and quality metrics on UMAP.

Fig. S4 spectrum.01320-26-s0004.pdf Spatial localization and enrichment analysis of clusters 1-3.

Table S1 spectrum.01320-26-s0005.xlsx Top 100 marker genes identified in transcriptional clusters 0, 1, 2, 3, 5, and 6.

ASM does not own the copyrights to Supplemental Material that may be linked to, or accessed through, an article. The authors have granted ASM a non-exclusive, world-wide license to publish the Supplemental Material files. Please contact the corresponding author directly for reuse.
