# Supplementary material for: TRPV1–STAT3 signaling as a driver of lethal pathology during pneumococcal nose-to-brain invasion
Source: Microbiol Spectr. 2026 Sep 15;14(10):e01320-26. doi: 10.1128/spectrum.01320-26 (PMC13689226; doi:10.1128/spectrum.01320-26)
Supplement: Supplementary file 1 [file spectrum.01320-26-s0003.pdf]

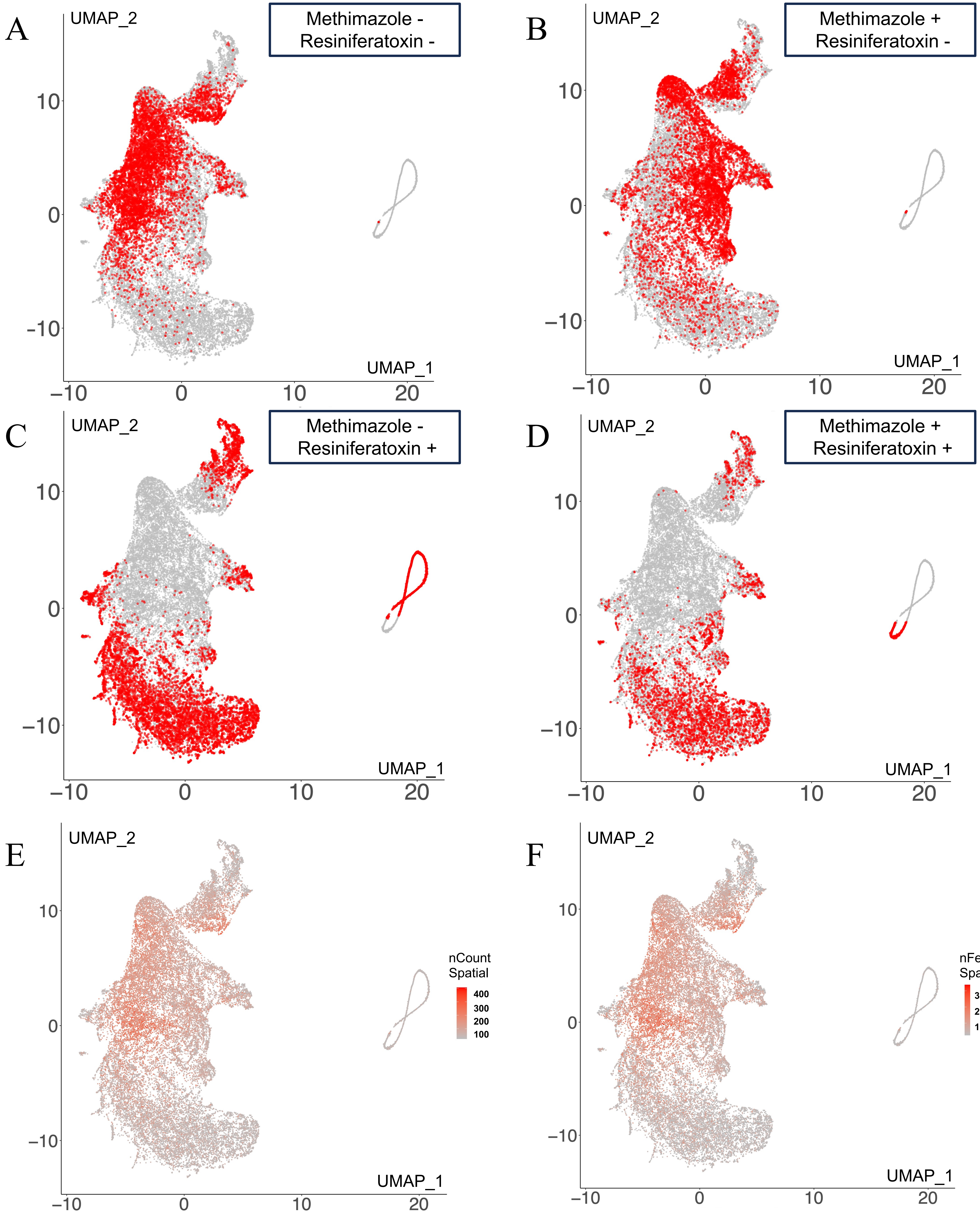

Supplementary Fig. 3. Distribution of spatial transcriptomic spots and quality metrics on UMAP
(A–D) UMAP projection showing the distribution of spatial transcriptomic spots from each group. Red dots indicate spots derived from each group, while gray dots representing all spots. Panels correspond to control (A), MZ (B), RTX (C), and MZ-RTX (D) groups. **(E)** Distribution of nCount_Spatial on the UMAP embedding. **(F)** Distribution of nFeature_Spatial on the UMAP embedding.
