# Supplementary material for: TRPV1–STAT3 signaling as a driver of lethal pathology during pneumococcal nose-to-brain invasion
Source: Microbiol Spectr. 2026 Sep 15;14(10):e01320-26. doi: 10.1128/spectrum.01320-26 (PMC13689226; doi:10.1128/spectrum.01320-26)
Supplement: Supplementary file 2 [file spectrum.01320-26-s0004.pdf]

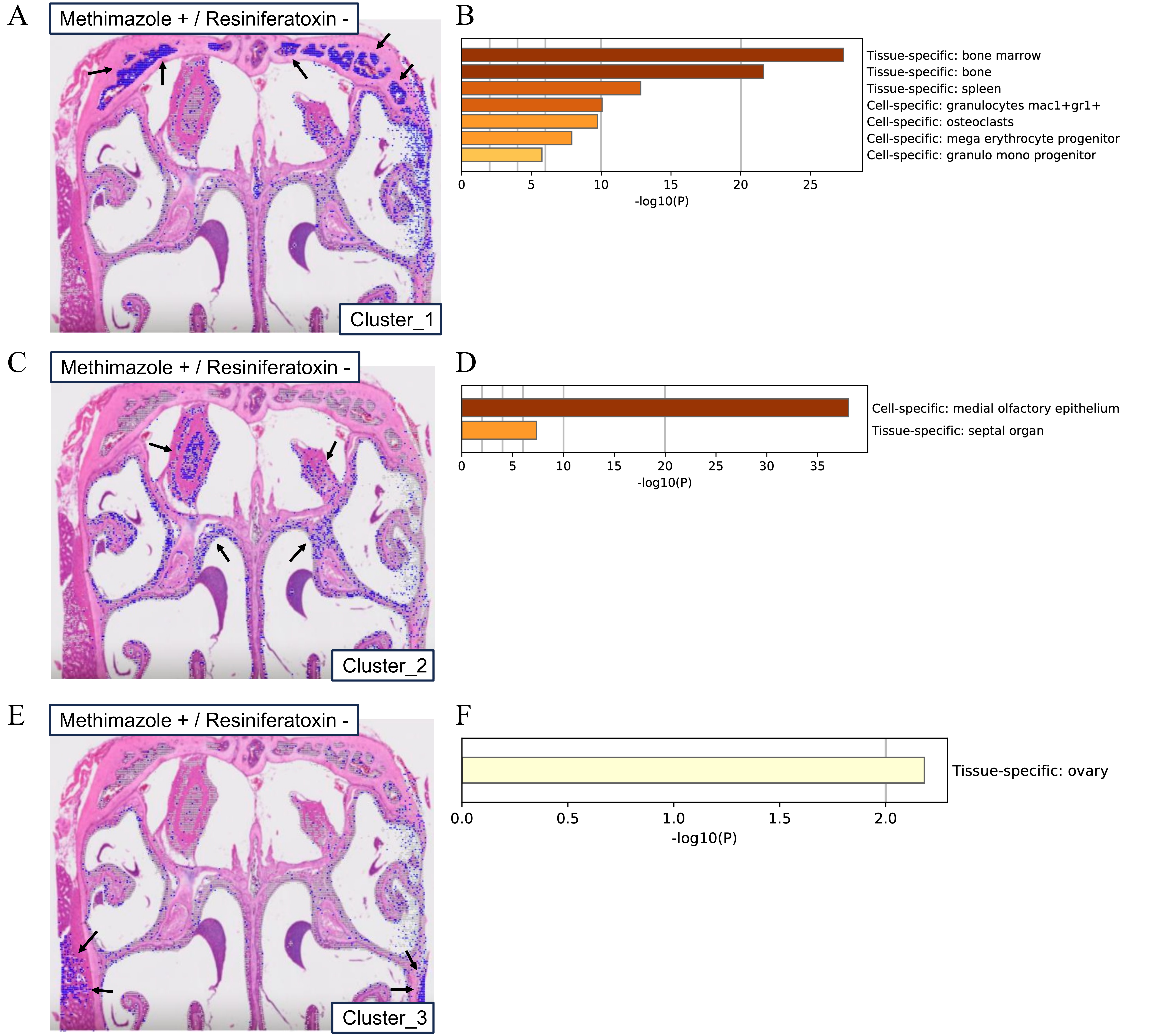

Supplementary Figure 4. Spatial localization and enrichment analysis of clusters 1–3

(A, C, E) Spatial localization of clusters 1, 2, and 3 on HE-stained coronal head sections from the MZ group. Colored dots represent spatial transcriptomic spots assigned to each cluster. Black arrows indicate representative regions enriched for each cluster. Cluster 1 was mainly localized within the cranial bone marrow (A), cluster 2 within the olfactory epithelium and olfactory bulb (C), and cluster 3 within the masticatory muscle (E).

(B, D, F) Gene ontology enrichment analysis of cluster-associated genes. Cluster 1 showed enrichment for vascular or hematopoietic/immune-associated signatures, including bone marrow, granulocyte, and osteoclast-related signatures (B). Cluster 2 showed enrichment for medial olfactory epithelium-associated signatures (D). Cluster 3 did not show biologically meaningful pathway or tissue enrichment relevant to the present study (F). Bars indicate statistical significance expressed as –log10(P).
